# Functional Characterization of Peroxiredoxins from the Human Protozoan Parasite *Giardia intestinalis*

**DOI:** 10.1371/journal.pntd.0002631

**Published:** 2014-01-09

**Authors:** Daniela Mastronicola, Micol Falabella, Fabrizio Testa, Leopoldo Paolo Pucillo, Miguel Teixeira, Paolo Sarti, Lígia M. Saraiva, Alessandro Giuffrè

**Affiliations:** 1 CNR Institute of Molecular Biology and Pathology, Rome, Italy; 2 Department of Biochemical Sciences and Istituto Pasteur – Fondazione Cenci Bolognetti, Sapienza University of Rome, Italy; 3 L.Spallanzani National Institute for Infectious Diseases, IRCCS, Rome, Italy; 4 Instituto de Tecnologia Quimica e Biológica, Universidade Nova de Lisboa, Oeiras, Portugal; Johns Hopkins Bloomberg School of Public Health, United States of America

## Abstract

The microaerophilic protozoan parasite *Giardia intestinalis*, causative of one of the most common human intestinal diseases worldwide, infects the mucosa of the proximal small intestine, where it has to cope with O_2_ and nitric oxide (NO). Elucidating the antioxidant defense system of this pathogen lacking catalase and other conventional antioxidant enzymes is thus important to unveil novel potential drug targets. Enzymes metabolizing O_2_, NO and superoxide anion (O_2_^−•^) have been recently reported for *Giardia*, but it is yet unknown how the parasite copes with H_2_O_2_ and peroxynitrite (ONOO^−^). *Giardia* encodes two yet uncharacterized 2-cys peroxiredoxins (Prxs), *Gi*Prx1a and *Gi*Prx1b. Peroxiredoxins are peroxidases implicated in virulence and drug resistance in several parasitic protozoa, able to protect from nitroxidative stress and repair oxidatively damaged molecules. *Gi*Prx1a and a truncated form of *Gi*Prx1b (delta*Gi*Prx1b) were expressed in *Escherichia coli*, purified and functionally characterized. Both Prxs effectively metabolize H_2_O_2_ and alkyl-hydroperoxides (cumyl- and tert-butyl-hydroperoxide) in the presence of NADPH and *E. coli* thioredoxin reductase/thioredoxin as the reducing system. Stopped-flow experiments show that both proteins in the reduced state react with ONOO^−^ rapidly (*k* = 4×10^5^ M^−1^ s^−1^ and 2×10^5^ M^−1^ s^−1^ at 4°C, for *Gi*Prx1a and delta*Gi*Prx1b, respectively). Consistent with a protective role against oxidative stress, expression of *Gi*Prx1a (but not delta*Gi*Prx1b) is induced in parasitic cells exposed to air O_2_ for 24 h. Based on these results, *Gi*Prx1a and delta*Gi*Prx1b are suggested to play an important role in the antioxidant defense of *Giardia*, possibly contributing to pathogenesis.

## Authors Summary

*Giardia intestinalis* causes one of the most common human intestinal diseases worldwide, called giardiasis. This microorganism infects the small intestine where it has to cope with O_2_, nitric oxide (NO) and related reactive species that are toxic for *Giardia* as it lacks most of the conventional antioxidant enzymes. Understanding how this pathogen survives oxidative stress is thus important because it may help to identify novel drug targets to combat giardiasis. Some enzymes playing a role in the antioxidant defense of *Giardia* have been recently reported, but it is yet unknown how the parasite copes with two well-known oxidants, hydrogen peroxide (H_2_O_2_) and peroxynitrite (ONOO^−^). In this study, the Authors show that *Giardia* expresses two enzymes (called peroxiredoxins), yet uncharacterized, that are able not only to degrade both H_2_O_2_ and ONOO^−^, but also to repair damaged molecules (called hydroperoxides) that accumulate in the cell under oxidative stress conditions. These results are totally unprecedented because no enzymes with these types of functions have been reported for *Giardia* to date. If these two enzymes will prove to be essential for *Giardia* virulence in future studies, a new way will be paved towards the discovery of novel drugs to treat giardiasis.

## Introduction

*Giardia intestinalis* is the amitochondriate protist causing giardiasis, one of the most common human intestinal diseases worldwide, responsible for 280 million symptomatic infections per year [1], [2], [3], [4]. This early divergent parasite has a relatively simple life cycle, alternating between two forms: the oro-fecally transmitted cyst and the vegetative trophozoite, which causes the disease by proliferating attached to the mucosa of the proximal small intestine. In this location this microaerophilic pathogen lacking most of the conventional antioxidant enzymes (including catalase, superoxide dismutase (SOD) and glutathione peroxidase [5], [6]) has to cope with both O_2_ and nitric oxide (NO). O_2_ tension in the proximal small intestine indeed not only is higher than in distal tracts of the gut [7], [8], [9], but it also fluctuates over time, peaking at every meal in order to meet metabolic demand. Moreover, the fine microcirculatory vascular network perfusing the intestinal submucosa reportedly contributes to formation of a steep O_2_ gradient such that O_2_ tension declines from up to 80–100 mm Hg at the submucosa to near anoxia at the luminal midpoint [10]. Living attached to the intestinal mucosa, it is therefore likely that *Giardia* trophozoites are physiologically exposed to fairly high O_2_ levels, as well as to the NO released by the NO-synthases in intestinal epithelial cells or derived from reduction of dietary nitrate/nitrite (see [10], [11] and references therein). Elucidating the *Giardia* antioxidant defense system that enables parasite survival to oxidative and nitrosative stress conditions is thus important, particularly in the perspective of unveiling novel potential pharmacological targets.

Defense systems against O_2_, NO and superoxide anion (O_2_^−•^) have been recently identified in *Giardia* as: a flavodiiron protein that, like a previously characterized NADH oxidase [12], is able to convert O_2_ to H_2_O [13], [14]; an inducible flavohemoglobin able to aerobically metabolize NO to nitrate [15], [16] and, more recently, a superoxide reductase reducing O_2_^−•^ to hydrogen peroxide (H_2_O_2_) [17]. Identification of the H_2_O_2_-producing superoxide reductase is puzzling, because *Giardia* lacks catalase and no H_2_O_2_-metabolizing enzymes have been characterized in the parasite to date; a NADH peroxidase activity has been reported in membrane extracts of the parasite [5], but the enzyme responsible for this activity is as-yet unidentified. On the other hand, toxicity of H_2_O_2_ against *Giardia* trophozoites is well documented [18], [19], being associated with depletion of cellular thiols, inactivation of O_2_ consumption, loss of membrane potential and cell motility [20], prompt degradation of the flavodiiron protein [14] and induction of a peculiar programmed cell death [21].

Peroxiredoxins (Prxs) [22], [23] are ubiquitous cysteine-dependent peroxidases found from bacteria and archaea to mammals, able to reduce H_2_O_2_ to H_2_O. Based on their catalytic mechanism and the number of cysteine (Cys) residues participating in catalysis, these enzymes are named ‘1-Cys’, ‘2-Cys’ or ‘atypical 2-Cys’ Prx. In homodimeric 2-Cys Prxs, one Cys-SH residue (‘peroxidatic Cys’, C_p_) is specifically oxidized by H_2_O_2_ to sulfenic acid (Cys–SOH), which in turn reacts with another Cys–SH (‘resolving Cys’, C_r_) on the adjacent monomer to produce a disulfide bond. This bond is then eventually reduced by a thiol-based protein, such as thioredoxin (Trx), to restore the initial fully reduced state of the enzyme. Besides detoxifying H_2_O_2_, Prxs also play a role in oxidative damage repair due to their alkyl hydroperoxide-reducing activity [24], and are involved in the detoxification of peroxynitrite (ONOO^−^) [25]. This is a harmful species generated by the reaction of nitric oxide (NO) with O_2_^−•^ at diffusion-controlled rates, causing oxidation and/or nitration of many biomolecules, including proteins, nucleic acids, lipids and thiols [26]. Given its cytotoxicity, ONOO^−^ is a key effector produced by the host immune system to counteract microbial infections. Prxs are multifunctional proteins, playing a role not only in the defense from nitroxidative damage, but also in signal transduction [27], [28] and protein folding [29], as well as in inflammation, tissue repair and tumor progression in higher Eukaryotes [30]. Interestingly, in several parasitic protozoa Prxs have been shown to be implicated in virulence and drug resistance [31].

Crystallographic studies revealed that all Prxs exhibit a similar topology, with a central 5-stranded β sheet, 5 α-helices and a 2-stranded β hairpin (see [22] and references therein). The two cysteine residues essential for the catalytic activity (C_p_ and C_r_), are often found in a conserved Val-Cys-Pro motif: C_p_ at the N-terminal part of the α_2_ helix, at the bottom of a pocket surrounded by the three additional conserved residues (Pro, Thr and Arg), and C_r_ at the N-terminal region. Depending on the redox state, typical 2-Cys Prxs alternate between two quaternary structures, a homodimer in the oxidized state and larger oligomeric forms, typically (do)decamers, in the reduced state. The crystal structure of two typical 2-Cys Prxs from protozoan parasites have been solved, the mitochondrial Prx from *Plasmodium (P.) falciparum* in the oxidized state [32] and tryparedoxin peroxidase from *Trypanosoma (T.) cruzi* [33].

The genome of *G. intestinalis* (http://giardiadb.org/giardiadb/) encodes two typical 2-Cys Prxs homologues belonging to the Prx1 subfamily (according to the nomenclature in [31]): *Gi*Prx1a (ORFs 16076 and 14521) and *Gi*Prx1b (ORF 15383). The enzymes are yet uncharacterized, but in a recent transcriptomic investigation [34] the expression of *Gi*Prx1a has been shown to be stimulated in *Giardia* trophozoites upon interaction with rat intestinal epithelial cells, pointing to a role of the protein in pathogenesis. Here, we report a detailed characterization of both Prxs from *Giardia*, focusing on their ability to metabolize H_2_O_2_, alkyl-hydroperoxides and ONOO^−^, and their expression profile in parasitic cells in response to O_2_ exposure.

## Methods

### Materials

H_2_O_2_, NADPH, cumene hydroperoxide (CumOOH), tert-butylhydroperoxide (t-butylOOH), dithiothreitol (DTT), *E. coli* thioredoxin (Trx), *E. coli* thioredoxin reductase (TrxR) and bovine catalase were purchased from Sigma-Aldrich. Peroxynitrite (ONOO^−^) was purchased from Cayman.

### Cloning, expression and purification of *Gi*Prx1a and *Gi*Prx1b

The GL50803_16076 gene (coding for *Gi*Prx1a) was amplified from *G. intestinalis* genomic DNA (150 ng) by PCR using the Taq DNA Polymerase High Fidelity and the primers 5′ – GAGATGAATTCATATGCCCGTC – 3′ and 5′ – GATTTGAAAGCTTCCCTCCTG – 3′ with restriction sites for Nde1 and HindIII, respectively. Similarly, the GL50803_15383 was amplified using Vent polymerase (New England Biolabs) and the primers 5′ – CTGCATGCAGCATATGACAACC – 3′ and 5′ – GTTAATGGGAGCTCTTCACTTTG – 3′ with restriction sites for Nde1 and SacI, respectively. The GL50803_15383 gene (coding for *Gi*Prx1b) was amplified without the portion encoding the protein N-terminal domain (a putative 46 aa-long signal peptide), thus resulting in a truncated version of the protein (here denoted as ‘delta*Gi*Prx1b’), because attempts to obtain the full length protein in a stable soluble form were unsuccessful. The amplified genes were NcoI and XhoI cloned into the expression vector pET28a(+) (GENEART GmbH, Regensburg, Germany).

Transformed *E. coli* BL21-Gold (DE3) cells were grown aerobically at 37°C in LB medium supplemented with 30 µg mL^−1^ kanamycin. At OD_600 nm_∼0.6 , expression of the His-tagged proteins was induced with 0.4 mM isopropyl-β-D-1-thiogalactopiranoside, and the cells were further grown overnight at 20°C. The cells (typically ∼6 g from 2 L of culture) were harvested by centrifugation (20 min at 5000 g), resuspended in 70 ml of 50 mM Tris + 500 mM NaCl + 1 mM DTT, and lysed by sonication. After centrifugation (30 min at 14000 g), the supernatant was loaded onto a His-Trap affinity column (Amersham). The recombinant His-tagged protein (*Gi*Prx1a or delta*Gi*Prx1b) was then eluted with 400 mM imidazole, which was then removed by gel filtration chromatography.

Concentration of the isolated proteins was determined using the bicinchoninic acid assay and their purity assessed by sodium dodecyl sulphate polyacrylamide gel electrophoresis (SDS-PAGE).

### Reaction of *Gi*Prxs with H_2_O_2_ and alkyl-hydroperoxides

The reaction of *Gi*Prx1a and delta*Gi*Prx1b with H_2_O_2_, t-butylOOH or CumOOH was investigated at 25°C, using a Jasco V-650 spectrophotometer. The peroxydatic activity of the two proteins (2–4 µM each) was measured in 50 mM HEPES + 1 mM EDTA + 100 mM NaCl pH = 7.2, following the oxidation of NADPH (100–200 µM) at 340 nm in the presence of *E. coli* TrxR (0.4–12 U mL^−1^) and *E. coli* Trx (10–80 µM), acting as artificial electron donors for the Prxs, and 100 µM H_2_O_2_ (or t-butylOOH or CumOOH) as the final electron accepting substrate. The rates were corrected for the rate of NADPH oxidation measured in the presence of all reactants prior to the addition of Prxs. The concentration of NADPH and H_2_O_2_ was determined photometrically using the extinction coefficients ε_340 nm_ = 6.22×10^3^ M^−1^ cm^−1^ and ε_240 nm_ = 43.6 M^−1^ cm^−1^ [35], respectively.

### Reaction of *Gi*Prxs with peroxynitrite

The reaction of reduced *Gi*Prx1a and delta*Gi*Prx1b with peroxynitrite (ONOO^−^, ε_302 nm_ = 1.67×10^3^ M^−1^ cm^−1^ [36]) was investigated by time-resolved spectroscopy, using a thermostated stopped-flow instrument (DX.17MV, Applied Photophysics, Leatherhead, UK) equipped with a 1-cm path length observation chamber. Experiments were carried out according to the ‘initial rate approach’ described in [37]. Briefly, each of the two Prxs in 100 mM phosphate buffer pH = 7.0+0.2 mM diethylenetriamine pentaacetic acid was reduced by 2 h-incubation with 10 mM DTT at room-temperature. Prior to the experiment, DTT was removed by concentration/dilution cycles and each protein at increasing concentration (from 10 to 150 µM) was anaerobically mixed at 4°C with a solution of 40 µM ONOO^−^ in 10 mM NaOH. According to [37], initial rates of ONOO^−^ decomposition were obtained from the absorption decrease measured at 310 nm, using ε = 1600 M^−1^ cm^−1^.

### Cultures of *G. intestinalis* trophozoites

Trophozoites of the *G. intestinalis* strain WB clone C6 (ATCC 50803) were cultured axenically at 37°C in modified Diamond's TYI-S-33 medium as previously described [38]. The medium was supplemented with 10% heat-inactivated bovine serum (Invitrogen) and 0.05% bovine bile (Sigma). Cultures were inoculated in 25 cm^2^-flasks, filled to 90% of their total volume in order to attain low-O_2_ tension conditions. Trophozoites were transferred every 48 h into fresh medium when cells became confluent. For preparation of the inocula, trophozoites were detached by chilling the cultures on ice for 30 min.

### Immunoblotting assays

For immunoblotting assays, *Giardia* trophozoites were plated in sterile 6-well plates at a density of 1×10^6^ cells mL^−1^ in a volume of 3 mL medium/well. Incubation was performed at 37°C allowing the plates to equilibrate for 24 h either under anaerobic conditions (Anaerocult A minisystem, Merck) or with air (atmospheric O_2_ level), in the presence or absence of 120 U mL^−1^ catalase.

After incubation, trophozoites were detached on ice for 30 minutes, collected by centrifugation and lysed (lysis buffer C3228, Sigma). After total protein content determination by the bicinchoninic acid assay, cell extracts (20 µg protein/lane) were subjected to SDS–PAGE and proteins blotted onto a polyvinylidene difluoride (PVDF) membrane (Immobilon pSQ, Merck). Blots were then incubated with rabbit polyclonal antibodies raised against *Gi*Prx1a or delta*Gi*Prx1b (Davids Biotechnologie GmbH), followed by incubation with alkaline peroxidase-conjugated secondary antibodies (NA934, GE Healthcare) and detection by enhanced chemiluminescence (ECL kit RPN2132, GE Healthcare). In these assays, using either the anti-*Gi*Prx1a or the anti-delta*Gi*Prx1b antibodies was irrelevant, because in dot-blot experiments each of the two antibodies showed cross-reactivity with both *Gi*Prxs.

### Real time qPCR

To quantify gene expression by real-time quantitative polymerase chain reaction (qPCR), based on genomic information (http://giardiadb.org/giardiadb/), primers specific for the *G. intestinalis* ORFs coding for *Gi*Prx1a (GL50803_16076), *Gi*Prx1b (GL50803_15383) and the housekeeping ribosomal small subunit protein S26 (GL50803_17364) were designed with the software Primer3 (v. 0.4.0, http://frodo.wi.mit.edu/primer3/) (Table 1) and purchased from Primm (Milan, Italy). Due to the very high nucleotide sequence identity between the two genes, primers designed for the 16076 gene are expected to target also the 14521 gene (Figure S1).

**Table 1:** Primers sequence (5′→3′) .

Rib S26_left	GAACATCGTTCGCTGTCAGA
Rib S26_right	GATGGAGCACGACACACAGT
*Gi*Prx1a (16076/14521)_left	GCCAAGCGTAAGCTCTCTGA
*Gi*Prx1a (16076/14521)_right	ACCAGGCGTAGTGGCTGTAG
*Gi*Prx1b (15383)_left	TGAGAAGTTTGGCGACACAG
*Gi*Prx1b (15383)_right	GGTGAAGTCGGCTGGATAGA

As for immunoblotting assays, trophozoites were plated in sterile 6-well plates at a density of 1×10^6^ cells mL^−1^ in a volume of 3 mL medium/well, and incubated up to 24 h under air, with or without 120 U mL^−1^ catalase, or under anaerobic conditions (Anaerocult A minisystem, Merck). After incubation, trophozoites were detached on ice for 30 minutes, washed in sterile phosphate buffered saline (PBS), collected by centrifugation and lysed with the lysis buffer from the High Pure RNA Isolation Kit from Roche. A DNaseI digestion step was included to remove possible residual genomic DNA. Quality of the extracted RNA was assessed by 1% agarose gel electrophoresis and from the A_260 nm_/A_280 nm_ absorbance ratio. According to the manufacturer's instructions, 5 µg total RNA were used for synthesizing the first cDNA strand, using the Thermo Scientific kit #K1641, with both random and oligo(dT)_18_ primers. qPCR assays were carried out with a Mx3000P Q-PCR System instrument (Agilent Technologies), using 2 µL cDNA as template, the Thermo Scientific Maxima SYBR Green qPCR Master Mix (2×) (# K0252), and the primers at a final concentration of 300 nM.

DNA amplification was carried out by running 40 cycles, each cycle including a denaturation (95°C, 15 s), an annealing (55°C, 30 s) and an extension (72°C, 30 s) step. Melting curve analysis was performed at the end of each run. No DNA was amplified if no reverse transcription was carried out, thus confirming the lack of contaminant genomic DNA in the samples. Each experimental condition was assayed in triplicate in at least three independent experiments. Data were normalized to the mRNA levels of the ribosomal small subunit protein S26 (ORF 17364).

### Data analysis

Multiple sequence alignments were obtained using Clustal Omega [39], [40]. Data from time-resolved spectroscopy were analysed using the software MATLAB (MathWorks, South Natick, MA, USA). Densitometric analysis of blotted membranes was carried out with the software Image J (http://imagej.nih.gov/ij). Statistical significance of the data was determined using the Student *t*-test in Microsoft Excel; all *P values* correspond to two-sided sample *t-*test assuming unequal variances. Graphs were generating using the software Origin7. The reported error bars represent the standard error of the mean (SEM).

### Accession numbers (UniProtKB)

*Gi*Prx1b_15383 (**A8BU8**); *Gi*Prx1a_16076 (**A8BYC4**); *Gi*Prx1a_14521 (**A8B338**); Prx1a from *T. brucei* (**Q71SQ4**); Prx1a from *T. cruzi* (**O96763**); Prx1m from *T. cruzi* (**O79469**); Prx from *P. berghei* (**Q4Z2P4**); Prx from *L.* donovani (**Q9BP39**); Prx from *Trichomonas vaginalis* (**Q8IEV2**); Prx from *E. dispar* (**Q9NL90**); Prx from *E. histolytica* (**B1N5A8**); Prx from *Plasmodium falciparum* (**Q8IL80**); TrxR from *G. intestinalis* (**E2RU27**), putative Trx from *G. intestinalis* (**A8B5E9**).

## Results

In the genome of *G. intestinalis* assemblage A (GL50803), there are three genes annotated as putatively coding for 2-Cys Prxs (Figure S1): the almost identical 16076 and 14521 genes (coding for *Gi*Prx1a), and the 15383 gene (coding for *Gi*Prx1b). Due to the very high (∼99%) nucleotide sequence identity between the 16076 and 14521 genes, only the proteins encoded by the 16076 (*Gi*Prx1a) and 15383 (*Gi*Prx1b) genes were considered for this study. Amino acid sequence analysis (Figure S2) shows that both *Gi*Prx1a and *Gi*Prx1b share significant similarities with Prxs from other protozoan parasites and, as expected, retain the two catalytically relevant, redox active cysteines (namely, Cys58 and Cys174 in *Gi*Prx1a and Cys95 and Cys219 in *Gi*Prx1b). At variance with *Gi*Prx1a, *Gi*Prx1b exhibits at its N-terminus 46 residues that are recognized by the software SignalP-4.1 [41] as a signal peptide, with a cleavage site between positions 15 and 16 (Figures S2); this suggests that *Gi*Prx1b may have a different intracellular localization as compared to *Gi*Prx1a, or it may even represent a secretory protein. Attempts to obtain the recombinant full-length *Gi*Prx1b protein in a stable soluble form were unsuccessful. The protein was therefore herein characterized as a truncated form (delta*Gi*Prx1b) devoid of the N-terminal signal peptide.

The recombinant His-tagged proteins *Gi*Prx1a and delta*Gi*Prx1b were purified to homogeneity by affinity chromatography with a typical yield of >20 mg protein per g of cells. As determined by SDS-PAGE (see a representative gel in Figure S3), both proteins were purified as single polypeptide chains with a molecular mass ∼23 KDa, consistent with the values calculated from amino acid sequences (22,540 and 22,690 Da for *Gi*Prx1a and delta*Gi*Prx1b, respectively).

### H_2_O_2_- and alkylhydroperoxide-reductase activity of *Gi*Prxs

**Figure 1:**
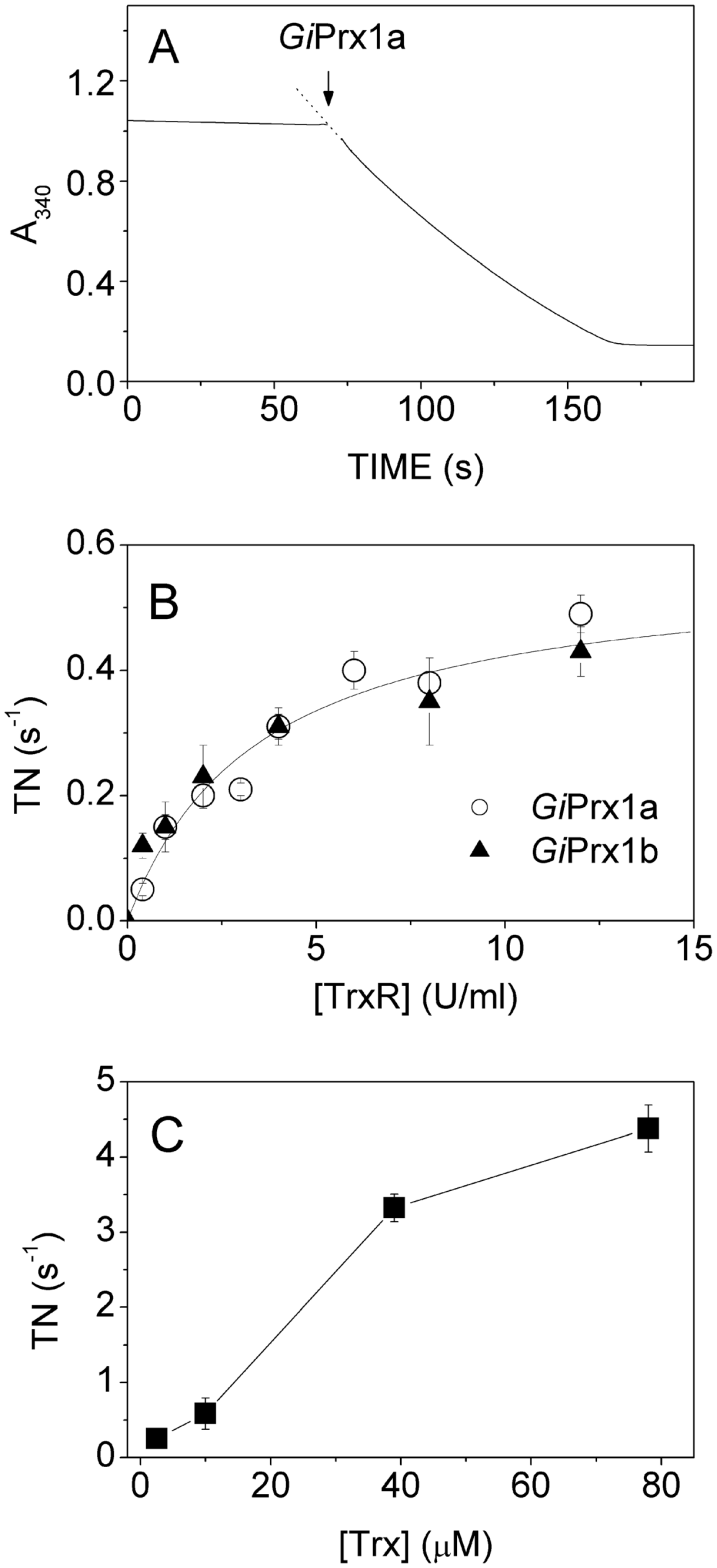
H_2_O_2_-reductase activity of *Gi*Prxs. A) NADPH oxidation measured in the presence of 200 µM NADPH, 4 U mL^−1^ *E. coli* TrxR, 10 µM *E. coli* Trx and 100 µM H_2_O_2_, following the addition of 2 µM *Gi*Prx1a. B) Turnover number (TN) measured in the presence of 10 µM *E. coli* Trx at increasing concentrations of *E. coli* TrxR (mean ±1 SEM, n≥4 for *Gi*Prx1a and n≥3 for delta*Gi*Prx1b). C) Activity of *Gi*Prx1a measured in the presence of 12 or 24 U mL^−1^ *E. coli* TrxR at increasing concentrations of *E. coli* Trx (mean ±1 SEM, n = 4).

The ability of the isolated recombinant *Gi*Prx1a and delta*Gi*Prx1b proteins to reduce H_2_O_2_ or alkyl-hydroperoxides, such as CumOOH and tButylOOH, was tested spectrophotometrically at 25°C by measuring NADPH oxidation at 340 nm, upon addition of the enzymes to a solution containing *E. coli* TrxR and Trx to mediate electron transfer to the Prxs. A representative assay with H_2_O_2_ as the final electron accepting substrate is reported in Fig. 1A, which shows a clear peroxidase catalytic activity of the Prxs. The activity, determined from the initial rate of NADPH oxidation, is underestimated due to the limited efficiency of the *E. coli* chimeric reducing system in providing electrons to *Giardia* Prxs. This has been confirmed in experiments in which the concentration of either TrxR (Fig. 1B) or Trx (Fig. 1C) from *E. coli* was systematically increased. Keeping [Trx] constant at 10 µM and increasing the concentration of TrxR, the H_2_O_2_-reductase activity of both *Gi*Prx1a and delta*Gi*Prx1b progressively increased saturating at >15 U mL^−1^ TrxR (Fig. 1B), while at high TrxR concentration (12 or 24 U mL^−1^) the apparent turnover number of *Gi*Prx1a increased almost proportionally with the concentration of Trx up to 4 s^−1^ (Fig. 1C). In these assays, where H_2_O_2_ was used at a maximal concentration of 100 µM, a progressive but slight inactivation of the recombinant Prxs was observed during the reaction, likely due to protein cysteine(s) hyperoxidation [42]. From these data, we conclude that both *Gi*Prx1a and delta*Gi*Prx1b can effectively reduce H_2_O_2_ with similar rates. V_max_, however, could not be determined because the concentrations of *E. coli* TrxR and Trx used in the assays did not prove to be saturating for *Gi*Prxs.

**Figure 2:**
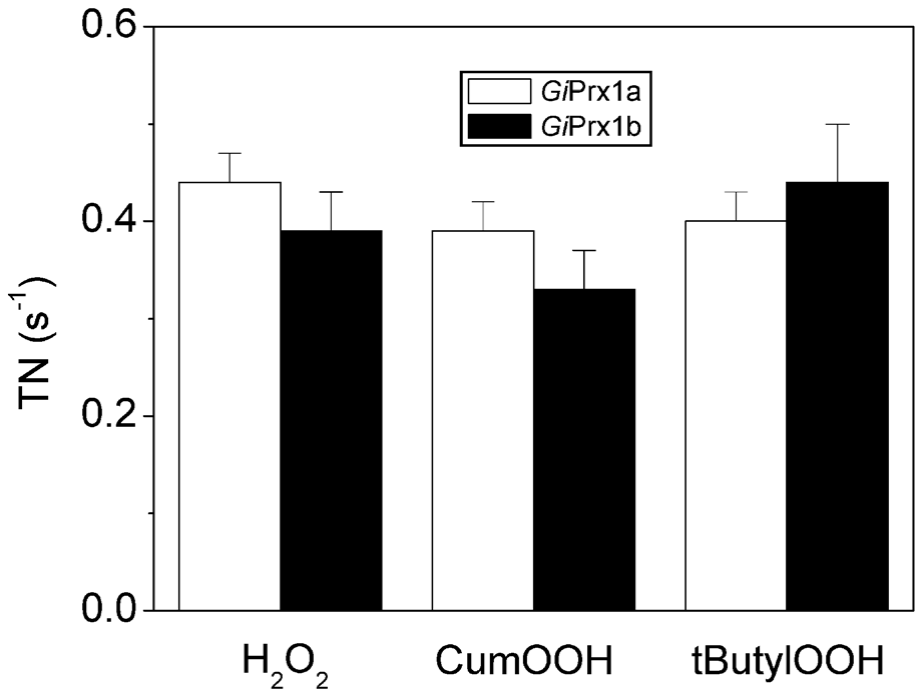
Alkyl-hydroperoxide reductase activity of *Gi*Prxs. Turnover number (TN) measured in the presence of 200 µM NADPH, 4 to 12 U mL^−1^ *E. coli* TrxR, 10 µM *E. coli* Trx and 100 µM CumOOH or 100 µM t-butylOOH, as compared to the activity measured with 100 µM H_2_O_2_ under otherwise identical conditions (mean ±1 SEM, n≥8 for *Gi*Prx1a and n≥6 for delta*Gi*Prx1b).

Importantly, by keeping the concentration of NADPH, TrxR and Trx constant and replacing H_2_O_2_ with CumOOH or tButylOOH, we could show that, under the experimental conditions tested, both *Gi*Prx1a and delta*Gi*Prx1b reduce alkyl-hydroperoxides at least as efficiently as they metabolize H_2_O_2_ (Fig. 2).

### Reaction of *Gi*Prxs with peroxynitrite

**Figure 3:**
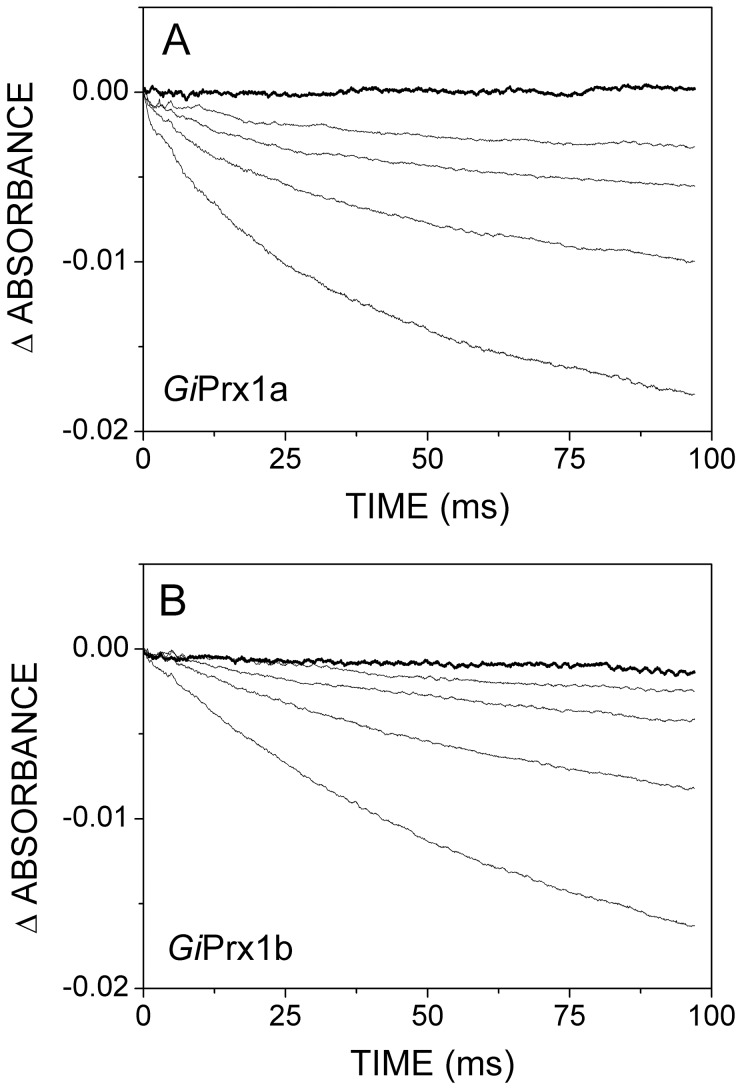
Reaction of reduced *Gi*Prxs with ONOO^−^. Absorption changes measured at 310 nm after anaerobically mixing in the stopped-flow apparatus a solution of ONOO^−^ with degassed buffer alone (thick lines) or containing reduced G*i*Prx1a (A) or deltaG*i*Prx1b (B) at increasing concentrations (thin lines). T  =  4°C. Concentrations after mixing: [ONOO^−^]  =  20 µM; [G*i*Prx1a]  =  0, 7.8, 15.5, 31 and 62 µM (from top to bottom); [deltaG*i*Prx1b]  =  0, 4.7, 9.4, 13.8 and 37.5 µM (from top to bottom).

The reaction of DTT-reduced *Gi*Prx1a and delta*Gi*Prx1b with peroxynitrite (ONOO^−^) was investigated by stopped-flow spectroscopy, following the experimental protocol described in [37]. The experimental temperature was set at 4°C so to slow down the reaction and measure the initial rate of the reaction with higher accuracy. Upon rapidly mixing under anaerobic conditions ONOO^−^ with either of the two Prxs in the reduced state, over the first 100 ms a fast consumption of ONOO^−^ was detected as a rapid absorption decrease at 310 nm; a representative experiment is reported in Fig. 3. The observed fast ONOO^−^ consumption is protein-mediated and dependent on the redox state of the Prxs; consistently, the reaction does not take place over the same time window (100 ms) in the absence of the proteins (thick lines in Fig. 3) or following their oxidation by an excess of H_2_O_2_ (not shown).

**Figure 4:**
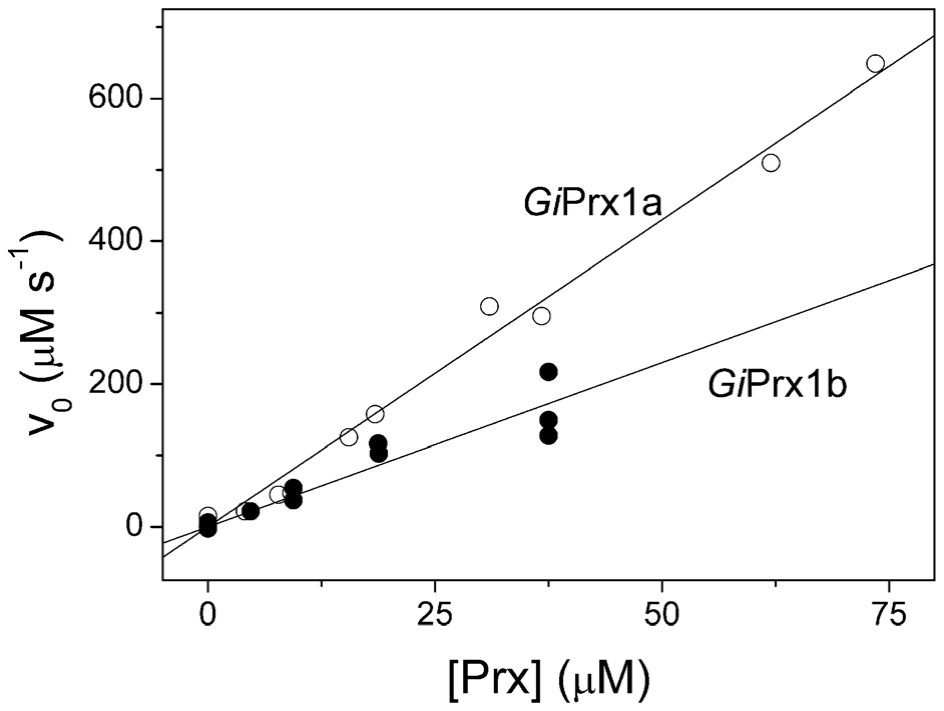
Initial rate of ONOO^−^ decomposition by *Gi*Prxs. Initial rates of ONOO^−^ consumption as a function of the *Gi*Prxs concentration. From linear regression analysis, the following second-order rate constants were determined: *k*∼4×10^5^ M^−1^ s^−1^ (*Gi*Prx1a) and *k*∼2×10^5^ M^−1^ (delta*Gi*Prx1b).

To estimate the second-order rate constant for the reaction of ONOO^−^ with reduced *Gi*Prx1a and delta*Gi*Prx1b, the kinetics of ONOO^−^ consumption was investigated at increasing concentrations of the two proteins. As expected, faster ONOO^−^ consumption was observed at higher protein concentrations (Fig. 3). In full agreement with previous data [37], the initial rate of the reaction was found to be proportional to the protein concentration (Fig. 4) and linear regression of the data allowed us to estimate the second-order rate constants *k*∼4×10^5^ M^−1^ s^−1^ and ∼2×10^5^ M^−1^ s^−1^ for *Gi*Prx1a and delta*Gi*Prx1b, respectively. ONOO^−^ decomposition is thus much faster in the presence of either of the two proteins than in their absence; for instance, the initial consumption rate of 20 µM ONOO^−^ increases by 70- or 35-fold in the presence of 20 µM reduced *Gi*Prx1a or delta*Gi*Prx1b, respectively. As internal control, we investigated the kinetics of the reaction of ONOO^−^ with free cysteine and found that, at pH = 7 and 4°C, the reaction proceeds with a second order rate constant *k*∼1×10^3^ M^−1^ s^−1^ (Figure S4). This rate constant, consistent with the one (*k*∼5.9×10^3^ M^−1^ s^−1^) previously measured at 37°C and pH = 7.4 by Radi et al. [43], is more than 100-fold smaller than the rate constant measured for the reaction of ONOO^−^ with *Gi*Prx1a or delta*Gi*Prx1b under otherwise identical experimental conditions.

### Effect of O_2_ on *Gi*Prxs expression

**Figure 5:**
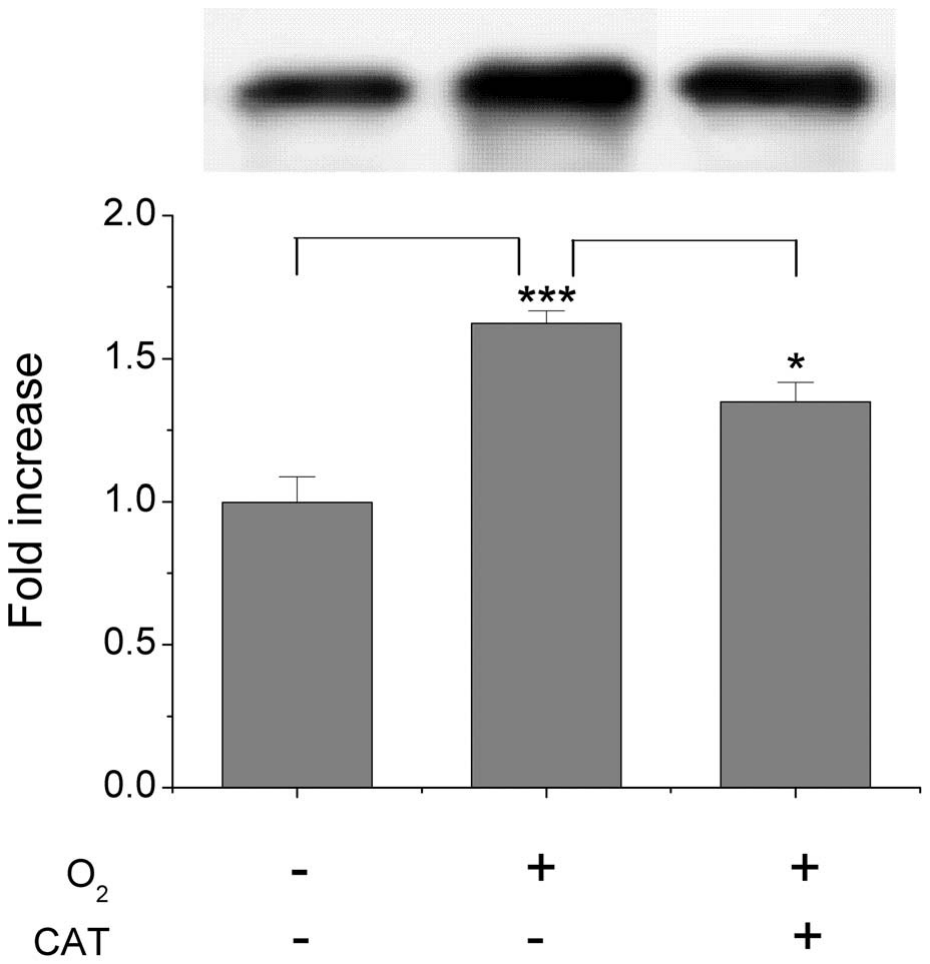
Immunodetection of *Gi*Prxs in parasitic cells: Effect of O_2_. Western blot analysis of *Gi*Prxs expression in trophozoites grown for 24 h in anaerobic standard conditions or under air, with or without 120 U mL^−1^ catalase. Densitometric data expressed with reference to the protein levels detected under anaerobic conditions (mean ±1 SEM, n≥5). ***P≤0.0002 vs anaerobic conditions. *P≤0.05 vs aerobic conditions w/o catalase.

Expression of the two Prxs in *Giardia* trophozoites was investigated both by immunoblotting and real time qPCR. Immunoblotting assays, however, did not allow us to discriminate between *Gi*Prx1b and *Gi*Prx1a (produced by either the 16076 or the almost identical 14521 gene), because polyclonal antibodies raised against either of two proteins cross-reacted with both Prxs (not shown). As shown in Fig. 5, Prxs can be immunodetected in trophozoites grown under standard anaerobic conditions and, interestingly, their expression is overall increased (∼1.6 fold) when parasitic cells are exposed for 24 h to air levels of O_2_. Notably, this effect is partly reverted by addition of catalase in the medium, pointing to a role of H_2_O_2_ in modulation of *Gi*Prxs expression.

**Figure 6:**
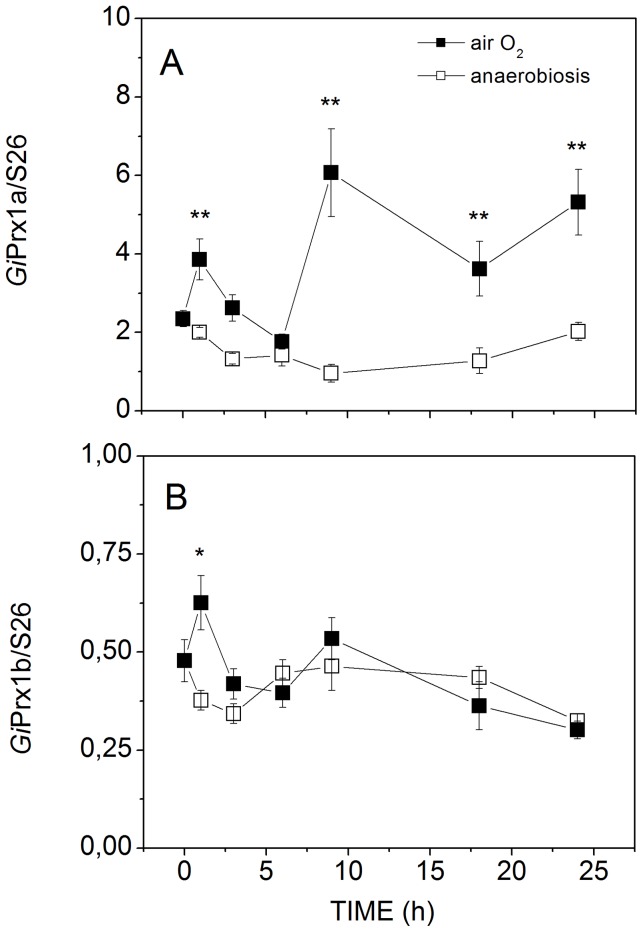
Effect of O_2_ on the transcriptional levels of *Gi*Prxs. Expression level of the genes coding for *Gi*Prx1a (A) and *Gi*Prx1b (B), evaluated by qPCR as a function of the incubation time of trophozoites with air O_2_ (closed symbols) or under anaerobic conditions (open symbols). Data (mean ±1 SEM, n≥3) were normalized to the mRNA level of the housekeeping ribosomal small subunit protein S26 (ORF 17364). *P<0.05 and **P<0.01 (aerobic vs anaerobic conditions at the same incubation time).

**Figure 7:**
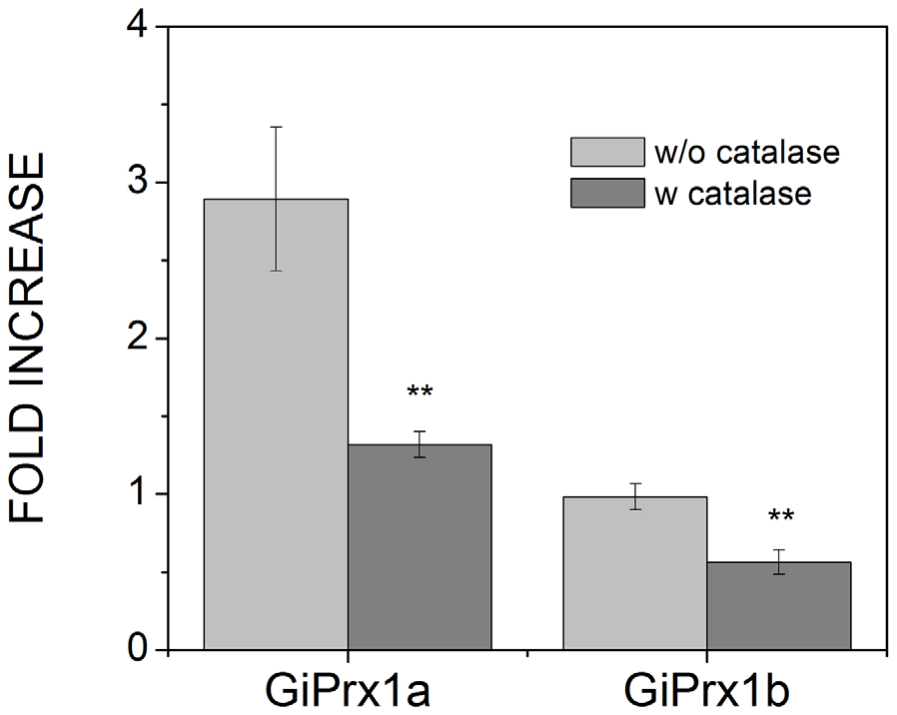
Effect of exogenous catalase on the transcriptional levels of *Gi*Prxs. Transcriptional levels of *Gi*Prx1a and *Gi*Prx1b measured in parasitic cells after 24 h-incubation with air, in the presence or absence of 120 U mL^−1^ catalase (mean ±1 SEM, n≥4). Data were normalized to the mRNA levels of the ribosomal small subunit protein S26 (ORF 17364), and expressed as the ratio to the expression levels detected in trophozoites grown under anaerobiosis for 24 h. **P<0.01.

In order to discriminate between the two Prxs, transcription of the genes encoding *Gi*Prx1a (16076 and 14521) or *Gi*Prx1b (15383) was individually analyzed by real time qPCR in parasitic cells, as a function of the incubation time (from 1 to 24 h) with air. As shown in Fig. 6, after normalization to the mRNA level of the housekeeping ribosomal small subunit protein S26, regardless of the presence or absence of O_2_, at any incubation time the mRNA of *Gi*Prx1a was found to be more abundant (at least 4 fold) than that one of *Gi*Prx1b. After 1 h-exposure to air O_2_, both Prxs showed slightly increased (∼2 fold) mRNA levels. However, at longer exposure times, the transcription profile of the two proteins was different: the mRNA level of *Gi*Prx1b constantly decreased, whereas at t ≥6 h the transcription of *Gi*Prx1a was stimulated, and after 24 h-exposure to air O_2_ the protein mRNA level was ∼3 fold higher than measured under anaerobic conditions. Interestingly, and in agreement with the immunoblotting analysis, in the presence of catalase scavenging H_2_O_2_, lower expression levels were detected for both *Gi*Prx1a and *Gi*Prx1b in parasitic cells exposed to air for 24 h (Fig. 7). In the light of the results obtained by real time qPCR, we conclude that *Gi*Prx1a is the Prx primarily detected by immunoblotting.

## Discussion

In several parasitic protozoa, Prxs have been shown to be implicated in virulence and drug resistance [31]. For instance, Prx1a from *T. brucei* has been validated as a drug target [44], and in *T. cruzi* the expression of two Prxs (*Tcru*Prx1a and *Tcru*Prx1m) was found to be up-regulated during infection, correlating with parasitic virulence [45]. Along the same line, in the invasive form of *Entamoeba (E.) histolytica*, the Prx expression level proved to be much higher than in the closely related *E. dispar* species, incapable of invasion [46]; even more revealing, the level of Prxs in non-virulent *E. histolytica* strains was shown to be lower than in the virulent ones [47], although in either strain the transcriptional levels did not change upon H_2_O_2_ or NO stress [48]. A Prx-deleted mutant of the mouse parasite *P. berghei* showed a reduced number of gametocytes [49] and oocysts [50], and more recently in *P. falciparum* PfTPx-1 was found to have a hypertermal-protective function, relevant for survival of the parasite in the human body after repeated incidences of fever [51]. Over and above these functions, overexpression of Prxs in parasitic protozoa has been also shown to enhance resistance to some drugs, namely to antimony in *Leishmania* (*L.*) donovani [52], to benznidazole in *T. cruzi* [53], [54] and to metronidazole in both *E. histolytica* [55] and *Trichomonas vaginalis* [56]. Finally, some Prxs appear to be also promising antigens for the development of new vaccines (see [31] and references therein), as exemplified by the Prx1 from *L. donovani* patented for such application (U.S. Patent 7795406). For all these reasons, Prxs are considered potential targets for the development of new antiparasitic treatments.

Despite the great body of information currently available on Prxs from parasitic protozoa [31], this is the first study focusing on the functional characterization of the two Prxs (*Gi*Prx1a and *Gi*Prx1b) identified in *G. intestinalis*. As a key observation, it is found that these proteins are both able to metabolize not only H_2_O_2_, but also the harmful ONOO^−^ and alkyl-hydroperoxide model compounds. These results are unprecedented for *G. intestinalis* because, to our knowledge, no enzymes from this parasite able to catalyze these physiologically relevant reactions have been characterized to date.

Prxs typically belong to an electron transfer chain that includes a NADPH-dependent thioredoxin-reductase (TrxR) and thioredoxin (Trx) as electron carrier. A TrxR has been previously identified in *Giardia*, purified and characterized [57]. However, attempts to measure the activity of this enzyme in crude extracts of the parasite in the presence of the putative *Giardia* Trx protein (encoded by the GL50803_3910 gene), recombinantly produced in *E. coli*, or its homologues from *E. histolytica* or *T. vaginalis*, were unsuccessful [58]. In the absence of the physiological redox partner of *Giardia* Prxs, the ability of *Gi*Prx1a and delta*Gi*Prx1b to turnover with H_2_O_2_ and alkyl-hydroperoxides was tested here following NADPH oxidation in the presence of the *E. coli* TrxR and Trx proteins (Figs. 1 and 2). While allowing measurements, this non-physiological chimeric reducing system even at the highest concentrations tested proved to rate-limit substrate consumption by the two Prxs (see Figs. 1B and 1C), thus preventing V_max_ measurements. Nevertheless, under identical experimental conditions the two Prxs exhibited the same apparent peroxidatic activity, regardless of the oxidizing substrate used in the assay (H_2_O_2_, CumOOH or tButylOOH, Fig. 2). Over time, a progressive slight inactivation of the two Prxs was observed as inferred from the non-linear time course of NADPH consumption measured after the addition of the enzymes to the reaction mixture (Fig. 1A). Such a slow activity decline did not revert upon re-addition of NADPH (not shown); in analogy with other Prxs, it was therefore interpreted as a progressive accumulation of the inactive form of the enzyme with hyperoxidized cysteine(s) [42].

Notably, both *Gi*Prxs are also highly reactive towards ONOO^−^ (Figs. 3 and 4). In alkaline solutions, ONOO^−^ is rather stable; otherwise, it decomposes rapidly (t_1/2_ = 1 s at pH 7.4, 37°C) upon protonation to peroxynitrous acid (ONOOH) (pKa = 6.8) [43]. In our assays, *Gi*Prx1a and delta*Gi*Prx1b were found to catalyze the consumption of ONOO^−^ with second-order rate constants *k*∼4×10^5^ M^−1^ s^−1^ and *k*∼2×10^5^ M^−1^ s^−1^, respectively (Fig. 4). These values are within the range of those published for Prxs from different microbial sources [37], taking into account that the published values were obtained at higher temperature (25°C or 37°C), and assuming the rates to double for every 10°C degrees increase in temperature. The occurrence of enzymes promptly degrading ONOO^−^ in *Giardia* is not only consistent with a previous study [59], in which ONOO^−^ was reported to kill parasitic cells only at high concentrations (apparent IC_50_∼3 mM), but also likely relevant for parasite survival *in vivo*. *Giardia* trophozoites are indeed known to express enzymes, such as DT-diaphorase, that by reaction with O_2_ generate O_2_^−•^ [60], a potential source of ONOO^−^ in the fairly NO-rich environment of the mucosa of the proximal small intestine. Moreover, *Giardia* is known to utilize arginine as energy source [61] and secrete an arginine-consuming enzyme, arginine deiminase, upon interaction with intestinal epithelial cells [62]. The reduced availability of arginine, however, can establish favorable conditions for the production of ONOO^−^ [63] by the NO-synthases, via the combined release of ROS and NO. In this regard, the combined action of arginine deiminase and Prxs represents a safe strategy for the parasite to counteract NO stress. In this context, it is worth mentioning that we are not aware of reports providing direct evidence for the production of ONOO^−^ in the human small intestine under physiological conditions. However, a basal level of nitrotyrosine (used as an indirect marker of ONOO^−^) has been reported in the small intestine of animal models [64], [65].

The effect of air O_2_ on the expression of the two *Gi*Prxs in *Giardia* trophozoites has been investigated here both by immunoblotting and qPCR. As shown in Fig. 5, *Gi*Prxs are overall already expressed to sufficiently high levels to be immunodetected in cells grown under standard anaerobic conditions, which may be consistent with Prxs being constitutively expressed to act as a first line defense against oxidative stress. Nonetheless, exposure of parasitic cells to air for 24 h caused a ∼50% increase in Prxs content, further suggesting a defense role against O_2_ toxicity. Consistently, qPCR experiments showed that in terms of mRNA levels *Gi*Prx1a is more abundant than *Gi*Prx1b and its transcription is further stimulated following cell exposure to air O_2_ (Fig. 6). Notably, as proved by addition of exogenous catalase, in both type of experiments (immunoblotting and qPCR) H_2_O_2_ appears to be responsible for the O_2_-mediated up-regulation of expression (Figs. 5 and 7). All together these data suggest an involvement of *Gi*Prxs both in the early and in the late phase of the response to oxidative stress, in agreement with the ability of these enzymes to detoxify nitroxidative stressors and repair oxidatively damaged molecules.

In conclusion, *Gi*Prxs are the first enzymatic defense system against peroxides, alkyl-hydroperoxides and ONOO^−^ having been characterized in *Giardia* as yet. Owing to their ability to protect from nitroxidative stress, these enzymes are likely involved in parasite survival *in vivo*, possibly playing a role in pathogenesis. No direct information supporting such a role is currently available, but in this regard it is interesting that recently *Gi*Prx1a has been found to be up-regulated upon interaction of *Giardia* trophozoites with intestinal epithelial cells [34]. Future work should aim at testing whether *Gi*Prxs are implicated in *Giardia* virulence, thus representing potential drug targets.

## Supporting Information

Figure S1 Sequence analysis of the genes coding for *Gi*Prxs. A) Alignment of the three gene sequences encoding *Gi*Prx1a and *Gi*Prx1b. Boxes highlight the sequences targeted by the primers used in the qPCR assays. B) Pairwise comparison in terms of % identity of the nucleotide sequences.
(DOC)

Figure S2 Amino acid sequence analysis of *Gi*Prx1a and *Gi*Prx1b. A) Multiple amino acid sequence alignment of the Prxs from *Giardia* and their homologs from other parasitic protozoa. UniProtKB accession numbers: *Gi*Prx1a_16076 (**A8BYC4**), *Gi*Prx1b_15383 (**A8BU8**), *Gi*Prx1a_14521 (**A8B338**), *Entamoeba (E.) histolytica* (**B1N5A8**), *E. dispar* (**Q9NL90**), *Trypanosoma (T.) brucei* (**Q71SQ4**), *Leishmania (L.) donovani* (**Q9BP39**), *T. cruzi* (**O79469**). The two conserved cysteine residues in the active site are indicated in yellow. Grey blocks represent conserved residues. B) Pairwise comparison in terms of % identity of the predicted amino acid sequences. C) Schematic drawing of *Gi*Prx1a and *Gi*Prx1b, with the two active site cysteines (C_p_ and C_r_) and the *Gi*Prx1b signal peptide highlighted in black and red, respectively.
(DOC)

Figure S3 4–12% SDS-PAGE analysis. Lane 1: molecular mass marker (Invitrogen). Lanes: 2–4: 0.4, 0,7 and 1.5 µg His-tagged purified *Gi*Prx1a.
(DOC)

Figure S4 Reaction of free cysteine with ONOO^−^. A) Absorption changes measured at 310 nm after anaerobically mixing in the stopped-flow apparatus a solution of ONOO^−^ with degassed buffer alone (dashed line) or containing free cysteine at increasing concentrations. Traces are shown with their best fit to single exponential decays. Buffer: 100 mM phosphate buffer pH = 7.0+0.2 mM diethylenetriamine pentaacetic acid. T = 4°C. Concentrations after mixing: [ONOO^−^] = 25 µM; [Cysteine] = 0.625, 1.25, 2.5 and 5 mM (from right to left). B) Observed rate constants as a function of the cysteine concentration. Linear regression analysis of the data yields a second-order rate constant *k*∼1×10^3^ M^−1^ s^−1^.
(DOC)
