# Supplementary material for: Functional Characterization of Peroxiredoxins from the Human Protozoan Parasite Giardia intestinalis
Source: PLoS Negl Trop Dis. 2014 Jan 9;8(1):e2631. doi: 10.1371/journal.pntd.0002631 (PMC3886907; doi:10.1371/journal.pntd.0002631)
Supplement: Figure S1 — Sequence analysis of the genes coding for GiPrxs. A) Alignment of the three gene sequences encoding GiPrx1a and GiPrx1b. Boxes highlight the sequences targeted by the primers used in the qPCR assays. B) Pairwise comparison in terms of % identity of the nucleotide sequences. (DOC) [file pntd.0002631.s001.doc]

**A**

*Gi*Prx1a_16076 ------------------------------------------------------------

*Gi*Prx1a_14521 ------------------------------------------------------------

*Gi*Prx1b_15383 ATGCTACTGCTGATATGTCTTATCTCGGTTGCCTTTGCGGCTGCTTGTACGCTGACCGTG 60

*Gi*Prx1a_16076 ---------------------------------------------------------ATG 3

*Gi*Prx1a_14521 ---------------------------------------------------------ATG 3

*Gi*Prx1b_15383 AATAAGAATACGTGCATACCTGATAAGTGTGAGAAGTTTGGCGACACAGAAGTCTGCATG 120

***

***Gi*Prx1b Forward**

*Gi*Prx1a_16076 C------------------CCGTCCCCATCCCCGGCACGCCCTGCCCCGACTTCGAGGTC 45

*Gi*Prx1a_14521 C------------------CCGTCCCCATCCCCGGCACGCCCTGCCCCGACTTCGAGGTC 45

GiPrx1b_15383 CAGCTCAAGACAACCAGTTCCGTTCCCATTCCAGGCACCCCTTGTCCCGACTTTGAAGTG 180

* **** ***** ** ***** ** ** ******** ** **

GiPrx1a_16076 GACGTCGTCACCCCTGAGCTTAAGTTCGCCAAGCGTAAGCTCTCTGACTACAAGGGCAAG 105

*Gi*Prx1a_14521 GACGTCGTCACCCCTGAGCTTAAGTTCGCCAAGCGCAAGCTCTCTGACTACAAGGGCAAG 105

*Gi*Prx1b_15383 GAGGTCCTTACCCCTGAACTCAAGTTTACTAAGCGTAAGCTTGCAGATTACAAGGGCAAA 240

** *** * ******** ** ***** * ***** ***** * ** ***********

***Gi*Prx1a Forward**

GiPrx1a_16076 TACCTGATTGTCTTCTTCTATCCGCTCGACTTCACCTTCGTCTGCCCCTCGGAGATCATC 165

*Gi*Prx1a_14521 TACCTGATTGTCTTCTTCTACCCGCTCGACTTCACCTTCGTCTGCCCCTCGGAGATCATC 165

*Gi*Prx1b_15383 TATCTGATCATCTTCTTCTATCCAGCCGACTTCACCTTTGTCTGTCCCTCTGAGATCATT 300

** ***** ********** ** ************ ***** ***** ********

***Gi*Prx1b Reverse**

GiPrx1a_16076 CACTTCTCGAACCTGGCTGAGCAGCTGAAGAAGAAGTGCAACGCGGAGATCATCATCGGC 225

*Gi*Prx1a_14521 CACTTCTCGAATCTGGCTGAGCAGCTGAAGAAGAAGTGCAACGCGGAGATCATCATCGGC 225

*Gi*Prx1b_15383 CATTTTTCCTCTATGGCTGAGCAACTGAAGAAGAAGTATAACACTGAGATTATCATTGGC 360

** ** ** ********** ************* *** * ***** ***** ***

GiPrx1a_16076 TCGACGGACTCCGTCTACAGCCACTACGCCTGGTGCCTCCAGGGCCAGAACGAGGGCGGC 285

*Gi*Prx1a_14521 TCGACGGACTCCGTCTACAGCCACTACGCCTGGTGCCTCCAGGGCCAGAACGAGGGCGGC 285

*Gi*Prx1b_15383 TCGACAGACACTGTTTATAGCCACCACGCTTGGTGCCTTCAGAATAAGACAGACGGGGGG 420

***** *** * ** ** ****** **** ******** *** *** ** ** **

***Gi*Prx1a Reverse**

*Gi*Prx1a_16076 ATCGGGACGTGCAAGTGCGACCTCTTCGCCGACACGAACCACAAGATGGCCCGCGACTTC 345

*Gi*Prx1a_14521 ATCGGGACGTGCAAGTGCGACCTCTTCGCCGACACGAACCACAAGATGGCCCGCGACTTC 345

*Gi*Prx1b_15383 ATCGGGCCCTGCAAATGTGATCTATTCGCCGACACGAATCATAAGATGGCACGTGATTTT 480

****** * ***** ** ** ** ************** ** ******** ** ** **

*Gi*Prx1a_16076 GGCGTTCTGGTCGAGGACGCCGGCATTGCGCTCCGCGGGATGTTCATCGTCTCTGACAAG 405

*Gi*Prx1a_14521 GGCGTTCTGGTCGAGGACGCCGGCATTGCGCTCCGCGGGATGTTCATCGTCTCTGACAAG 405

*Gi*Prx1b_15383 GGCATACTCGTTGAGGAAATGGGTCTTGCTCTGCGTGCCATGTTTATCGTCTCCGATAAA 540

*** * ** ** ***** ** **** ** ** * ***** ******** ** **

*Gi*Prx1a_16076 GGCGTCGTTCGCCACGTCACGATCAACGATCTTCCAGTGGGTCGCTCGGTGGAGGAGGCC 465

*Gi*Prx1a_14521 GGCGTCGTTCGCCACGTCACGATCAACGATCTTCCAGTGGGTCGCTCGGTGGAGGAGGCC 465

*Gi*Prx1b_15383 GGAATTGTACGCCATGTTACAATTAACGATTTTCCTGTGGGTCGCTCTGTCGAAGAAGCT 600

** * ** ***** ** ** ** ****** **** *********** ** ** ** **

*Gi*Prx1a_16076 ATGCGCCTGGTCCAGGCCTTCCAGTACGCTGATAAAACCGGCGGCGACATCCCGTGCGGC 525

*Gi*Prx1a_14521 ATGCGCCTGGTCCAGGCCTTCCAGTACGCTGATAAAACCGGCGGCGTCATCCCGTGCGGC 525

*Gi*Prx1b_15383 ATGCGTATGATTCAGGCCTTCCAGTACGCTGATAAAACCGGCGGTGTCATCCCGTGCGGC 660

***** ** * ******************************** * *************

*Gi*Prx1a_16076 TGGACCCCCGAGAAGAATGACACTATCATCCCCGACCCCGAGAAGAAGAAGGAGTACTTC 585

*Gi*Prx1a_14521 TGGACCCCCGAGAAGAACGACACTATCATCCCCGACCCCGAGAAGAAGAAGGAGTACTTC 585

*Gi*Prx1b_15383 TGGACTCCTGAGAAGAACGACTTCATTATTCCTGATCCCGAAAAGAAGAAGGAATACTTT 720

***** ** ******** *** ** ** ** ** ***** *********** *****

*Gi*Prx1a_16076 TCCAAGACGTTCAAAGAGTGA 606

*Gi*Prx1a_14521 TCCAAGACGTTCAAGAAGTAA 606

*Gi*Prx1b_15383 TCTAAGACCTTCACAAAGTGA 741

** ***** **** *** *

**B**

|  | ***Gi*Prx1a_16076** | ***Gi*Prx1a_14521** | ***Gi*Prx1b_15383** |
| --- | --- | --- | --- |
| ***Gi*Prx1a_16076** | 100 | 99 | 79 |
| ***Gi*Prx1a_14521** | 99 | 100 | 79 |
| ***Gi*Prx1b_15383** | 79 | 79 | 100 |

**Figure S1** *Sequence analysis of the genes coding for GiPrxs*

A) Alignment of the three gene sequences encoding *Gi*Prx1a and *Gi*Prx1b. Boxes highlight the sequences targeted by the primers used in the qPCR assays.

B) Pairwise comparison in terms of % identity of the nucleotide sequences.
