# Supplementary material for: Functional Characterization of Peroxiredoxins from the Human Protozoan Parasite Giardia intestinalis
Source: PLoS Negl Trop Dis. 2014 Jan 9;8(1):e2631. doi: 10.1371/journal.pntd.0002631 (PMC3886907; doi:10.1371/journal.pntd.0002631)
Supplement: Figure S2 — Amino acid sequence analysis of GiPrx1a and GiPrx1b. A) Multiple amino acid sequence alignment of the Prxs from Giardia and their homologs from other parasitic protozoa. UniProtKB accession numbers: GiPrx1a_16076 (A8BYC4), GiPrx1b_15383 (A8BU8), GiPrx1a_14521 (A8B338), Entamoeba (E.) histolytica (B1N5A8), E. dispar (Q9NL90), Trypanosoma (T.) brucei (Q71SQ4), Leishmania (L.) donovani (Q9BP39), T. cruzi (O79469). The two conserved cysteine residues in the active site are indicated in yellow. Grey blocks represent conserved residues. B) Pairwise comparison in terms of % identity of the predicted amino acid sequences. C) Schematic drawing of GiPrx1a and GiPrx1b, with the two active site cysteines (Cp and Cr) and the GiPrx1b signal peptide highlighted in black and red, respectively. (DOC) [file pntd.0002631.s002.doc]

**A**

**E. hystolitica** MSCNQQKECCKKECQEKECC----------KECCCPRIKAFKKFINTFEKAQIGKEAPEF 50

**E. dispar** MSCNQQKECCKKECQEKECCKEYCCKEYCKKECCCPRIKAFKKFMNTFEKAQIGKEAPEF 60

**T. vaginalis** --------------------------------------------------MLVGKPAPAF 10

**T. brucei** ---------------------------------------------MSCGDAKLNHPAPHF 15

**L. donovani** ---------------------------------------------MSCGNAKINCPAPPF 15

**T. cruzi** MFRRMAVTSLQKGLS---------------RRAFCNTLRLLNLDYQAYKTATVREAAPEW 45

***Gi*Prx1a_16076** -----------------------------------------------MPVPIPGTPCPDF 13

***Gi*Prx1a_14521** -----------------------------------------------MPVPIPGTPCPDF 13

***Gi*Prx1b_15383** --MLLLICLISVAFAAACTLTVNKNTCIPDKCEKFGDTEVCMQLKTTSSVPIPGTPCPDF 58

**E. hystolitica** KAPAYCPCGSIKEIDINEYKGKYVVLLFYPLDWTFVCPTEMIGYSELAGQLK-EINCEVI 109

**E. dispar** KAQAYCPCGSIKEIDINEYKGKYVVLLFYPLDWTFVCPTEMIGYSEVAGQLK-EINCEVI 119

**T. vaginalis** KGQAVFPDTDFKEVSLEQYKGKWLVLFSYPLDFTFVCPTEIIEFSNKYEEFK-KIGCEVL 69

**T. brucei** NEVALMPNGTFKKVDLASYRGKWVVLFFYPLDFTFVCPTEICQFSDRVKEFN-DVDCEVI 74

**L. donovani** EEVALMPNGSFKKISLAAYKGKWVVLFFYPLDFTFVCPTEIIAFSENVSRFN-ELNCEVL 74

**T. cruzi** AGKAVV-NGKIQDISLNDYKGKYVVLLFYPMDFTFVCPTEITAFSDAQAEFD-KINTQVV 103

***Gi*Prx1a_16076** EVDVVTPELKFAKRKLSDYKGKYLIVFFYPLDFTFVCPSEIIHFSNLAEQLKKKCNAEII 73

***Gi*Prx1a_14521** EVDVVTPELKFAKRKLSDYKGKYLIVFFYPLDFTFVCPSEIIHFSNLAEQLKKKCNAEII 73

***Gi*Prx1b_15383** EVEVLTPELKFTKRKLADYKGKYLIIFFYPADFTFVCPSEIIHFSSMAEQLKKKYNTEII 118

**E. hystolitica** GVSVDSVYCHQAWCEADKSKGGVGKLTFPLVSDIKRCISIKYGMLNVEAGIARRGYVIID 169

**E. dispar** GVSVDSVYCHQAWCEADKSKGGVGKLGFPLVSDIKRCISIKYGMLNVETGVSRRGYVIID 179

**T. vaginalis** GLSVDSVFTHLAWINTPRKEGGLGEIKYPLIGDLGAKIAKEYGFYMCEAGHTLRGTAIID 129

**T. brucei** ACSMDSEFSHLAWTNVERKKGGLGTMNIPILADKTKSIMKAYGVLKEEDGVAYRGLFIID 134

**L. donovani** ACSMDSEYAHLQWTLQDRKKGGLGAMAIPMLADKTKSIARAYGVLEEKQGVAYRGLFIID 134

**T. cruzi** AVSCDSQYSHLAWINTPRNKGGLGEMSIPVLSDLTKEIARDYGVLIEEQGISLRGLFIID 163

***Gi*Prx1a_16076** IGSTDSVYSHYAWCLQGQNEGGIGTCKCDLFADTNHKMARDFGVLVEDAGIALRGMFIVS 133

***Gi*Prx1a_14521** IGSTDSVYSHYAWCLQGQNEGGIGTCKCDLFADTNHKMARDFGVLVEDAGIALRGMFIVS 133

***Gi*Prx1b_15383**  IGSTDTVYSHHAWCLQNKTDGGIGPCKCDLFADTNHKMARDFGILVEEMGLALRAMFIVS 178

**E. hystolitica** DKGKVRYIQMNDDGIGRSTEETIRIVKAIQFSDEHGAVCPLNWK-PGKDTIEPTSDG-IK 227

**E. dispar** DKGKVRYIQMNDDGIGRSTEETIRIVKAIQFSEEHGAVCPINWK-AGKDTIEPTPEG-IK 237

**T. vaginalis** PEGIIRHVQMNHPDVGRNVDEILRLIKAYQFAAKHGEVCPAQWHGEGDLTIKPNPKA-SK 188

**T. brucei** PQQNLRQITINDLPVGRNVDETLRLVKAFQFVEKHGEVCPANWK-PGSKTMKADPNGSQD 193

**L. donovani** PNGMVRQITVNDMPVGRNVEEVLRLLEAFQFVEKHGEVCPANWK-KGAPTMKPEPKASVE 193

**T. cruzi** DKGILRHITVNDLPVGRNVEEVLRVVQAFQYVDKNGDVIPCNWR-PGKPTMKTEK---AN 219

***Gi*Prx1a_16076** DKGVVRHVTINDLPVGRSVEEAMRLVQAFQYADKTGGDIPCGWTPEKNDTIIPDPEK-KK 192

***Gi*Prx1a_14521** DKGVVRHVTINDLPVGRSVEEAMRLVQAFQYADKTGGVIPCGWTPEKNDTIIPDPEK-KK 192

***Gi*Prx1b_15383** DKGIVRHVTINDFPVGRSVEEAMRMIQAFQYADKTGGVIPCGWTPEKNDFIIPDPEK-KK 237

**E. hystolitic**a KYLTTLKQTR 237

**E. dispar** KYLTTH---- 243

**T. vaginalis** EYFGKANN-- 196

**T. brucei** -YFSSMN--- 199

**L. donovani** GYFSKQ---- 199

**T. cruzi** EYFEKNA--- 226

***Gi*Prx1a_16076** EYFSKTFKE- 201

***Gi*Prx1a_14521** EYFSKTFKK- 201

**GiPrx1b_15383** EYFSKTFTK- 246

.

**B**

| **Prxs** | ***Gi*Prx1a_16076** | **GiPrx1b_15383** | ***Gi*Prx1a_14521** |
| --- | --- | --- | --- |
| ***Gi*Prx1a_16076** | 100 | 84 | 99 |
| **GiPrx1b_15383** | 84 | 100 | 85 |
| ***Gi*Prx1a_14521** | 99 | 85 | 100 |
| **E. hystolitic**a | 40 | 32 | 41 |
| **E. dispar** | 38 | 33 | 39 |
| **T. vaginalis** | 44 | 41 | 44 |
| **T. brucei** | 40 | 38 | 41 |
| **L. donovani** | 41 | 40 | 42 |
| **T. cruzi** | 44 | 38 | 45 |

**C
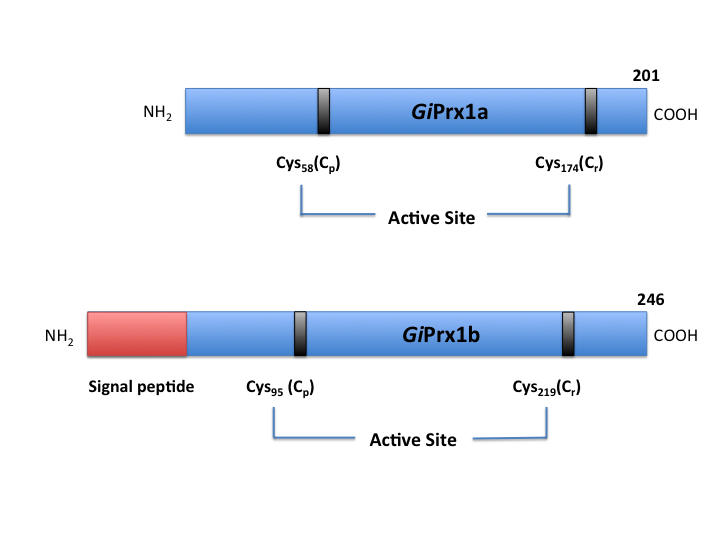
**

**Figure S2** *Amino acid sequence analysis of GiPrx1a and GiPrx1b*

A) Multiple amino acid sequence alignment of the Prxs from *Giardia* and their homologs from other parasitic protozoa. UniProtKB accession numbers: *Gi*Prx1a_16076 (**A8BYC4**), *Gi*Prx1b_15383 (**A8BU8**), *Gi*Prx1a_14521 (**A8B338**), *Entamoeba (E.) histolytica* (**B1N5A8**), *E. dispar* (**Q9NL90**), *Trypanosoma (T.) brucei* (**Q71SQ4**), *Leishmania (L.)* *donovani* (**Q9BP39**), *T. cruzi* (**O79469**). The two conserved cysteine residues in the active site are indicated in yellow. Grey blocks represent conserved residues.

B) Pairwise comparison in terms of % identity of the predicted amino acid sequences.

C) Schematic drawing of *Gi*Prx1a and *Gi*Prx1b, with the two active site cysteines (C_p_ and C_r_) and the *Gi*Prx1b signal peptide highlighted in black and red, respectively.
