# Supplementary material for: Functional Characterization of Peroxiredoxins from the Human Protozoan Parasite Giardia intestinalis
Source: PLoS Negl Trop Dis. 2014 Jan 9;8(1):e2631. doi: 10.1371/journal.pntd.0002631 (PMC3886907; doi:10.1371/journal.pntd.0002631)
Supplement: Figure S3 — 4–12% SDS-PAGE analysis. Lane 1: molecular mass marker (Invitrogen). Lanes: 2–4: 0.4, 0,7 and 1.5 µg His-tagged purified GiPrx1a. (DOC) [file pntd.0002631.s003.doc]

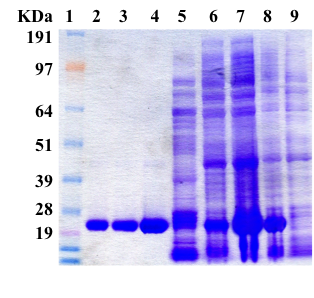


**Figure S3** *4-12% SDS-PAGE analysis*

Lane 1: molecular mass marker (Invitrogen). Lanes: 2 - 4: 0.4, 0,7 and 1.5 µg His-tagged purified *Gi*Prx1a.
