# Supplementary material for: Functional Characterization of Peroxiredoxins from the Human Protozoan Parasite Giardia intestinalis
Source: PLoS Negl Trop Dis. 2014 Jan 9;8(1):e2631. doi: 10.1371/journal.pntd.0002631 (PMC3886907; doi:10.1371/journal.pntd.0002631)
Supplement: Figure S4 — Reaction of free cysteine with ONOO−. A) Absorption changes measured at 310 nm after anaerobically mixing in the stopped-flow apparatus a solution of ONOO− with degassed buffer alone (dashed line) or containing free cysteine at increasing concentrations. Traces are shown with their best fit to single exponential decays. Buffer: 100 mM phosphate buffer pH = 7.0+0.2 mM diethylenetriamine pentaacetic acid. T = 4°C. Concentrations after mixing: [ONOO−] = 25 µM; [Cysteine] = 0.625, 1.25, 2.5 and 5 mM (from right to left). B) Observed rate constants as a function of the cysteine concentration. Linear regression analysis of the data yields a second-order rate constant k∼1×10^3^ M−1 s−1. (DOC) [file pntd.0002631.s004.doc]

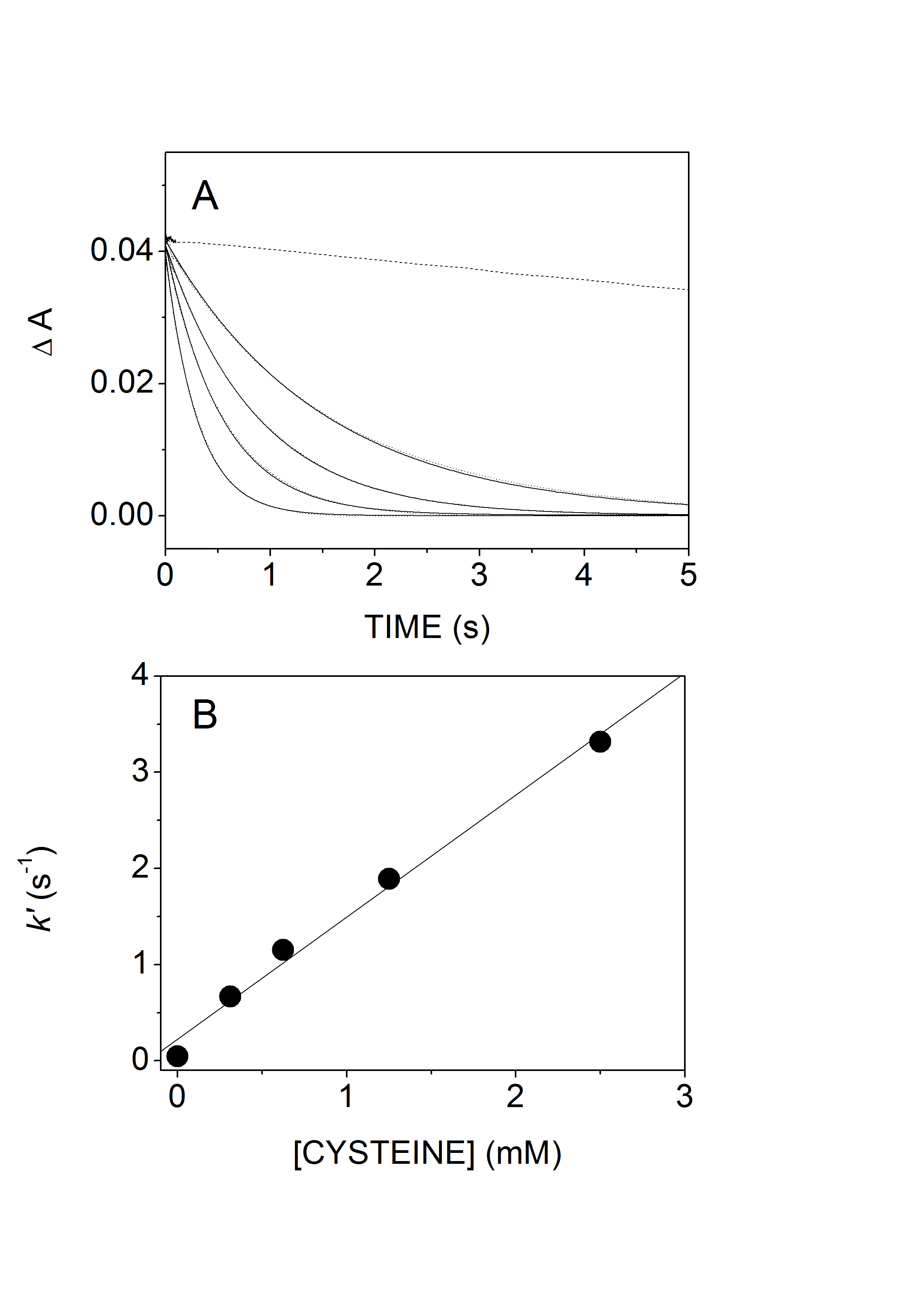


**Figure S4** *Reaction of free cysteine with ONOO^-^*

A) Absorption changes measured at 310 nm after anaerobically mixing in the stopped-flow apparatus a solution of ONOO^–^ with degassed buffer alone (dashed line) or containing free cysteine at increasing concentrations. Traces are shown with their best fit to single exponential decays. Buffer: 100 mM phosphate buffer pH = 7.0 + 0.2 mM diethylenetriamine pentaacetic acid. T = 4°C. Concentrations after mixing: [ONOO^–^] = 25 µM; [Cysteine] = 0.625, 1.25, 2.5 and 5 mM (from right to left).

B) Observed rate constants as a function of the cysteine concentration. Linear regression analysis of the data yields a second-order rate constant *k* ~ 1 x 10^3^ M^-1^ s^-1^.
